# The Na^+^ /H^+^ exchanger (NHE1) as a novel co-adjuvant target in paclitaxel therapy of triple-negative breast cancer cells

**DOI:** 10.18632/oncotarget.2860

**Published:** 2014-11-26

**Authors:** Schammim Ray Amith, Jodi Marie Wilkinson, Shairaz Baksh, Larry Fliegel

**Affiliations:** ^1^ Department of Biochemistry, University of Alberta, Edmonton, Alberta, Canada; ^2^ Department of Pediatrics, Biochemistry and Oncology, Alberta Inflammatory Bowel Disease Consortium, University of Alberta, Edmonton, Alberta, Canada

**Keywords:** NHE1, Paclitaxel, Triple-negative breast cancer, Metastasis, pH regulation

## Abstract

Dysregulation of Na^+^ /H^+^ exchanger isoform one (NHE1) activity is a hallmark of cells undergoing tumorigenesis and metastasis, the leading cause of patient mortality. The acidic tumor microenvironment is thought to facilitate the development of resistance to chemotherapy drugs and to promote extracellular matrix remodeling leading to metastasis. Here, we investigated NHE1 as a co-adjuvant target in paclitaxel chemotherapy of metastatic breast cancer. We generated a stable NHE1-knockout of the highly invasive, triple-negative, MDA-MB-231 breast cancer cells. The NHE1-knockout cells proliferated comparably to parental cells, but had markedly lower rates of migration and invasion *in vitro*. *In vivo* xenograft tumor growth in athymic nude mice was also dramatically decreased compared to parental MDA-MB-231 cells. Loss of NHE1 expression also increased the susceptibility of knockout cells to paclitaxel-mediated cell death. NHE1 inhibition, in combination with paclitaxel, resulted in a dramatic decrease in viability, and migratory and invasive potential of triple-negative breast cancer cells, but not in hormone receptor-positive, luminal MCF7 cells. Our data suggest that NHE1 is critical in triple-negative breast cancer metastasis, and its chemical inhibition boosts the efficacy of paclitaxel *in vitro*, highlighting NHE1 as a novel, potential co-adjuvant target in breast cancer chemotherapy.

## INTRODUCTION

In normal and neoplastic cells, the maintenance of pH homeostasis is chiefly regulated by the Na^+^ /H^+^ exchanger isoform one (NHE1). NHE1 is a ubiquitously expressed, integral plasma membrane protein comprised of a membrane-traversing domain that facilitates ion flux, and a cytoplasmic C-terminal domain that regulates exchanger activity. Ion flux occurs with the electroneutral exchange of one intracellular proton for one extracellular sodium ion, and is driven by the transmembrane Na^+^ gradient. In conditions of low intracellular pH (pH_i_), excess protons allosterically activate NHE1, facilitating proton extrusion and a return to homeostatic pH_i_. NHE1 activity therefore maintains pH_i_ and has been shown to play an important role in the regulation of cell volume and shape, while also promoting cell growth, proliferation, differentiation, and apoptosis [1]. NHE1 is present on the nuclear membranes of various cell types including aortic and liver tissues, and cardiomyocytes, suggesting that it is also involved in the modulation of intranuclear pH [2]. Regulation of NHE1 activity is thus critical and tightly controlled by multiple signaling pathways (e.g. MAPK, ERK1/2), and initiated by growth factors or hormonal stimulation that either directly or indirectly affect the phosphorylation state of its C-terminal domain. In addition, several intracellular proteins and lipids (e.g. PIP_2_, CHP1/2/3, actin binding proteins ezrin-radixin-moesin, calmodulin, carbonic anhydrase II) regulate Na^+^ /H^+^ exchange via interaction with the C-terminal tail of NHE1 [3]. Whether all these protein and lipid associations are retained in breast cancer cells is not clear [4].

In neoplastic cells, NHE1 activity is of special importance. The dysregulation of NHE1 activity is now considered a hallmark of cells undergoing tumorigenesis. NHE1 becomes constitutively active in the transformed phenotype, resulting in a reversal of the normal pH gradient in many cancer cells. This raises pH_i_ with a concomitant decrease in extracellular pH. The low extracellular pH (pH_e_) and decreased vascularization associated with rapid tumor growth also stimulate directed cell migration and invasion, resulting in the metastatic dissemination of tumor cells to sites distant from the primary tumor [5]. Importantly, the acidic extracellular microenvironment stimulates extracellular protease activity, extracellular matrix remodeling and cell invasion [6]. It may also contribute to the development of multidrug resistance of tumor cells to many chemotherapy drugs, which also hinders immune rejection of these tumors [7]. Weakly basic chemotherapy agents (e.g. doxorubicin) are likely protonated in acidic pH_e_, which impedes their entrance into cells and prevents them from reaching their intracellular molecular targets [8, 9]. In mice, xenograft tumors of lowly invasive MCF7 breast cancer cells were much more sensitive to doxorubicin at an extracellular pH of 7.4 than 6.8 *in vivo* [10]. Paclitaxel, which has a complex structure that includes both an acidic and basic domain, is also less cytotoxic at low pH_e_ [11]. To date, there is no evidence to suggest that pharmacological inhibitors of NHE1 could be effective chemotherapy agents in humans [12]. However, it stands to reason that manipulating the tumor microenvironment through the modulation of NHE1 activity could aid in chemotherapy treatment strategies in a co-adjuvant manner post-surgical intervention or, alternatively, in a co-neoadjuvant manner prior to surgery.

The initial development of NHE1-specific inhibitors was driven by the need to counter the adverse effects of excessive exchanger activity in the mammalian myocardium. Amiloride, a potassium-sparing diuretic that has been used clinically, is a NHE inhibitor [13, 14]. Several other drugs have since been developed and investigated in terms of their increased selectivity and potency towards NHE1 inhibition [15]. Testing these NHE1 inhibitors for their anti-cancer properties is ongoing [16]. The two major families of these compounds are: the pyrazine derivatives (e.g. 5-(N,N-hexamethylene)amiloride), 5-(N,N-dimethyl)amiloride, 5-(N-ethyl-N-isopropyl-amiloride)), and the benzoylguanidines (e.g. cariporide, eniporide, HOE-694) [13]. The successful use of amiloride as an anti-cancer therapy in animal models was recently reviewed [14].

Here, we propose that, since pH regulation is pivotal in the switch from the normal to the neoplastic to the metastatic phenotype of cancer cells, that inhibition of NHE1 can be used as a target to increase the efficacy of anti-cancer drugs. Recent studies have lent credence to this hypothesis. One study examined MCF7 breast cancer cells, representative of the estrogen receptor-positive luminal subtype of breast cancer. NHE1 knockdown or inhibition with 5-(N-ethyl-N-isopropyl) amiloride sensitized these cells to apoptosis induced by cisplatin [17].

Triple-negative breast cancer is a heterogeneous disease that accounts for 10-20% of all metastatic breast cancers. Triple-negative breast cancer lacks the expression of estrogen and progesterone receptors, and human epithelial growth factor 2 receptors (HER2; also known as ErbB2), and shares characteristics with basal-like, claudin-low, and BRCA1-related breast cancer. It is most commonly diagnosed in younger women (< 50 years) and ultimately results in poor prognosis [18]. To date, no targeted therapies exist for the treatment of metastatic triple-negative breast cancer other than surgery and cytotoxic chemotherapy, primarily with taxanes (e.g. paclitaxel) or anthracyclines (e.g. doxorubicin) [19]. In this study, we investigated NHE1 as a target for adjuvant therapy in highly invasive, triple-negative breast cancer cells. We used specific NHE1 inhibitors, HMA [5-(N,N-hexamethylene) amiloride)], representative of the pyrazine class of amiloride derivatives, and EMD87580 [2-methyl-4,5-di-(methylsulfonyl)-benzoyl-guanidine)], representative of the benzoylguanidines, to increase the susceptibility of triple-negative breast cancer cells to paclitaxel. Paclitaxel belongs to the taxane group of pharmaceuticals that was introduced into the clinical treatment of breast and ovarian cancer in the 1990s [20]. It is still considered the most effective treatment option for breast cancer patients and is US-FDA approved as a second line chemotherapy for those with advanced metastatic disease [21]. We report that low-dose paclitaxel-mediated cell death is increased by the simultaneous administration of either EMD87580 or HMA in triple-negative breast cancer cells. Furthermore, we validate the importance of NHE1 function by generating an NHE1-knockout cell line (231-KO) for comparison with the parental MDA-MB-231 cells that endogenously express NHE1. The 231-KO cells showed markedly less xenograft tumor growth than the parental MDA-MB-231 cells over time. Taken together, our data show, for the first time, that the chemical inhibition or loss of NHE1 expression in invasive, metastatic triple-negative breast cancer cell lines enhances their susceptibility to paclitaxel-mediated cell death. This study enforces the idea that NHE1 may be key in the development of novel, tumor microenvironment-targeted chemotherapeutic strategies for the treatment of triple-negative breast cancers.

## RESULTS

### Na^+^ /H^+^ exchange activity, but not NHE1 expression, is elevated in MDA-MB-231 (231-WT) cells in tumor mimetic conditions

**Figure 1:**
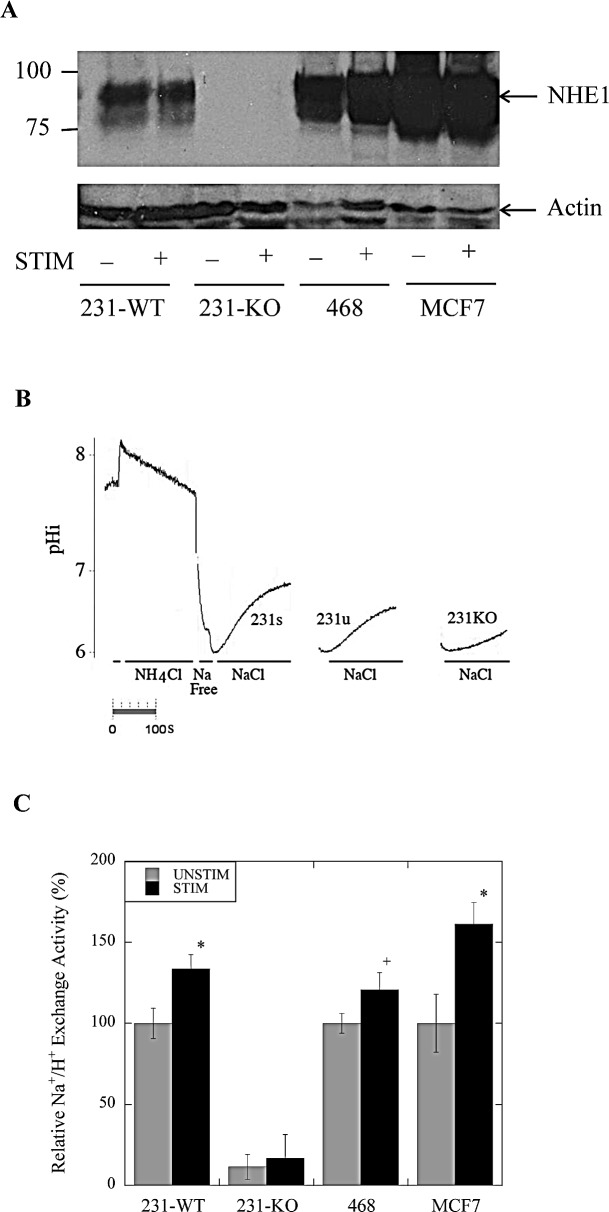
Na/H exchanger expression and activity in parental MDA-MB-231 (231-WT) and NHE1-knockout cells (231-KO) A, Western blot analysis of NHE1 expression in 231-WT, 231-KO, MDA-MB-468 and MCF7 cells probed with anti-NHE1 antibody. B, Changes in intracellular pH (pH_i_) in response to acid loading. Examples of traces illustrating the recovery from an acute acid load. Intracellular pH was examined in cells that were transiently acidified using ammonium chloride. Periods of NH_4_Cl, NaCl and Na-free solution are indicated. An entire example of the recovery is indicated for stimulated 231-WT cells (231s). An example of recovery is indicated for unstimulated 231-WT cells (231u) and MDA-MB-231 NHE1-knockout cells (231-KO). Fluorescence of BCECF, a pH-sensitive indicator dye, was used to record and quantify changes in pH_i_ post-acute acid load induced by ammonium chloride. NHE1 activity was calculated from the slope of the first 20 sec of recovery from acidification and was expressed as ΔpH/sec. C, Relative rate of Na^+^/H^+^ exchange activity of 231-KO cells relative to 231-WT cells, and in MDA-MB-468 and MCF7 cells. Values of ΔpH/sec from 231-WT and 231-KO cells were normalized to the unstimulated control values for 231-WT cells. Data for MCF7 and MDA-MB-468 were normalized to their control unstimulated values respectively. No discernible exchanger activity was detected in the knockout cells. Background drift and buffering capacity were not significantly different between cell types. In stimulated conditions (0.2% serum), NHE1 activity is significantly increased in 231-WT, MDA-MB-468 and MCF7 cells [*P<0.001, ^+^ P<0.01, N=8].

To examine the role of NHE1 in tumor cells we made a knockout of the protein in the parental MDA-MB-231 cells (231-WT). Western blot analysis was used to examine endogenous expression of NHE1 in the NHE1-knockout (231-KO), compared to the 231-WT cells, MCF7 and MDA-MB-468 cells. NHE1 was consistently observed as a band at approximately 100 kDa in multiple lysate preparations (Fig. 1A). A second smaller band was also observed and is typical of a partially or de-glycosylated NHE1 protein. As expected, the stable NHE1-knockout (231-KO) cells showed no NHE1 protein expression. We also compared the level of NHE1 protein in either stimulated (low-serum (0.2%) media) or unstimulated (10%-serum supplemented media) conditions as previously described [22]. When the level of NHE1 was quantitatively compared to that of actin, there was no up-regulation of NHE1 expression in any cell type under stimulated conditions (N=5, see Fig. 1 for example).

We examined the NHE1 activity of the tumor cell lines in stimulated or unstimulated conditions (Fig. 1B, C). As expected, stable NHE1-knockout MDA-MB-231 (231-KO) cells showed no demonstrable Na^+^ /H^+^ exchanger activity either in stimulated or unstimulated conditions. When we examined the relative Na^+^ /H^+^ exchange activity of MDA-MB-231 and MCF7 (P<0.001, N=8), and MDA-MB-468 (P<0.01, N=8) breast cancer cells, we found that it was significantly higher in cells that were serum-starved for 24 hr. prior to measurement of pH_i_ (Fig. 1B, C).

**Figure 2:**
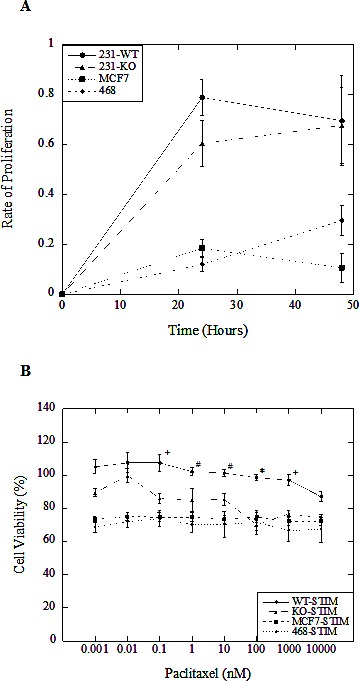
Characterization of NHE1-knockout (231-KO) MDA-MB-231 cells A, B, Cell proliferation and viability in response to increasing paclitaxel doses was assessed by spectrophotometric analysis of MTT (3-(4,5-dimethylthiazol-2-yl)-2,5-diphenyltetrazolium bromide) absorbance at 570 nm, with background subtraction at 630 nm. All data are presented as a ratio of mean OD values at indicated time points for each cell type relative to 0 hr. A, Proliferation of 231-WT and 231-KO cells. In stimulated (0.2% serum) conditions, proliferation of knockout cells is not significantly different from parental MDA-MB-231 cells. However, both 231-WT and 231-KO cells are more proliferative over 48 hr. compared to MDA-MB-468 (468) and MCF7 cells. B, Effect of paclitaxel on cell viability of 231-WT and 231-KO cells. Paclitaxel was significantly more cytotoxic to NHE1-knockout cells compared to parental MDA-MB-231 cells at concentrations of 0.1 nM and higher in stimulated conditions over 24 hours [*P<0.001, ^+^ P<0.01, ^#^ P<0.05, N=3]. In contrast, stimulated MDA-MB-468 and MCF7 cells did not show any changes in viability dependent on paclitaxel concentration.

To further characterize differences between parental and NHE1-knockout MDA-MB-231 cells, we studied cell proliferation in unstimulated and stimulated conditions over 48 hr. In serum-deprived conditions typical of the tumor microenvironment, both 231-WT and 231-KO cells were more proliferative compared to MCF7 and MDA-MB-468 cells over 48 hr. (Fig. 2A; P<0.001, N=5). There was no difference in proliferation rates between stimulated 231-WT and 231-KO cells. All cell types had similar rates of proliferation in unstimulated conditions (data not shown). We also examined the effect of increasing concentrations of paclitaxel on cell viability. Interestingly, stimulated 231-KO cells appeared to be more susceptible to paclitaxel-mediated cytotoxicity at 0.1 nM and higher concentrations (P<0.05 to P<0.001, N=3) compared to 231-WT cells (Fig. 2B). In addition, there were no significant changes in the viability of MCF7 and MDA-MB-468 cells in response to paclitaxel at the tested concentrations. In other experiments, we examined the effect of NHE1 inhibitors on cell viability. EMD87580 was not cytotoxic to cells at up to 100 μM, but high concentrations of HMA (100 μM) resulted in less than 10% viability in all cell types (data not shown).

### Knockout of NHE1 inhibits the growth of MDA-MB-231-driven subcutaneous tumors *in vivo* in athymic nude mice

**Figure 3:**
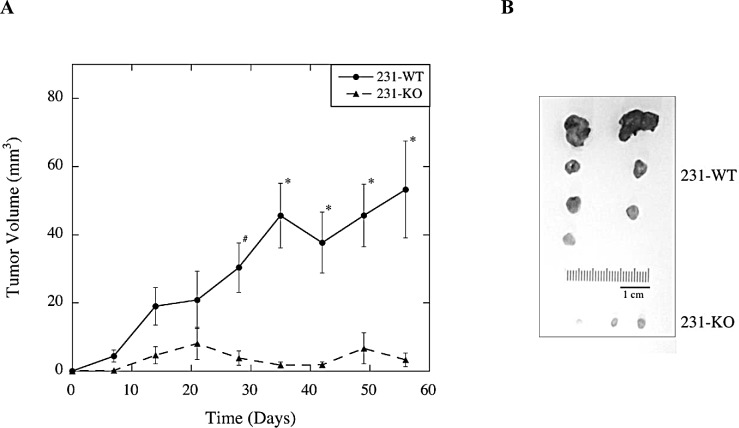
Effect of knocking out NHE1 on MDA-MB-231 xenograft tumor growth in female athymic nude mice A, Average tumor volume over time in mice subcutaneously injected with either 231-WT or 231-KO cells. Suspensions of 231-WT and 231-KO cells in Matrigel were subcutaneously injected into the right and left dorsal flanks of female athymic nude mice to determine their tumor-promoting potential. Tumor growth was monitored weekly and mice were euthanized at Day 60. Tumor volume was calculated based on the volume of a sphere (V = (4/3)πr^3^), or the length and width of the tumor mass (V = 0.5*x* X 2*y*, where *x* is the length and *y* is the width of the tumor mass). There were 4 mice per group: 7 of 8, and 3 of 8, tumor xenografts developed in the wild type and knockout group respectively. B, Excised tumors from all mice. 231-WT xenografted cells developed into significantly larger, heterogeneous tumors than the 231-KO cells starting at Day 28 [^#^ P<0.05, N=8], and showed a marked increase tumor growth after Day 35 [*P<0.01, N=8]. No significant tumor growth was observed in the knockout group.

To assess the tumor-promoting potential of MDA-MB-231 cells in relation to NHE1 expression, tumor growth of both 231-WT and 231-KO cells was examined as xenografts in athymic nude mice over 60 days. As shown in Fig. 3A, tumors derived from parental MDA-MB-231 cells (N=8) increased in volume over time, while the growth of 231-KO tumors was minimal, or not at all, with small tumors formed in only 3 of 8 injection sites. At 28 days, the observed differences in tumor growth were significant (P<0.05). At 35 days and onwards, growth of 231-WT tumors was markedly increased (P<0.001), but no difference was observed in 231-KO tumor growth. While heterogeneity of excised tumors was observed within the 231-WT group, the importance of NHE1 in tumor-promotion was evident from the lack of tumor formation with the 231-KO cells (Fig. 3B).

### NHE1 inhibition potentiates the effect of paclitaxel on cell viability

**Figure 4:**
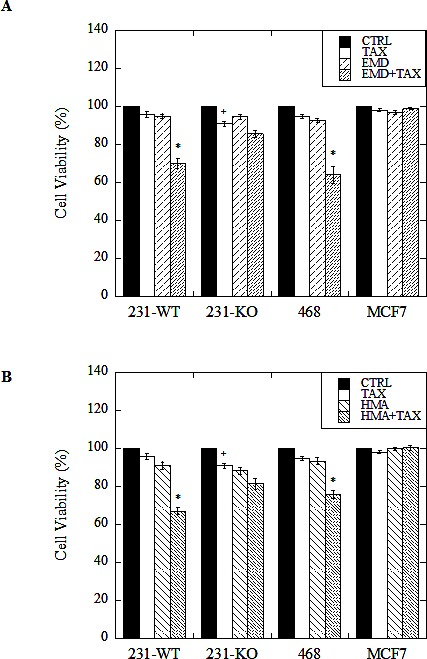
Effect of paclitaxel in combination with NHE1 inhibitors on cell viability of wild type (231-WT) and NHE1-knockout (231-KO) MDA-MB-231 cells, and MDA-MB-468 (468) and MCF7 cells Cells were treated for 24 hours in reduced (0.2%) serum media (stimulated conditions), with 1 nM paclitaxel (TAX), 10 μM EMD87580 (EMD) (A), or 10 nM HMA (HMA) (B), or with both paclitaxel and EMD87580 (ET, A), or paclitaxel and HMA (HT, B) [*P<0.001, ^+^ P<0.01, N=5]. Media only control cells (CTRL) were left untreated. Viability was assessed by the catalytic conversion of yellow MTT (3-(4,5-dimethylthiazol-2-yl)-2,5-diphenyltetrazolium bromide) to purple formazan in live cells. Spectrophotometric quantitation of this colorimetric change was recorded as the optical density of the sample at 570 nm, with background subtraction at 630 nm. All data are presented as a ratio of sample means over mean control values for each treatment for each cell type. In 231-KO cells, a significant loss of viability is observed when cells are treated with paclitaxel alone [^+^ P<0.01, N=5]. In stimulated conditions, both triple-negative MDA-MB-231 and MDA-MB-468 cells showed significantly reduced viability when treated with paclitaxel in the presence of NHE1 inhibitors EMD87580 or HMA [*P<0.001, N=5]. MCF7 cell viability, however, was unaffected by drug treatments.

As noted above, we found that stimulated 231-WT cells have elevated Na^+^ /H^+^ exchanger activity independent of changes in protein expression. We hypothesized that with NHE1 inhibition they would be more susceptible to paclitaxel-induced cell death because of a dependence on this elevated rate of Na^+^ /H^+^ exchange. We indeed found this to be the case. The viability of stimulated 231-WT cells was significantly decreased (P<0.001, N=5) when cells were treated with 1 nM paclitaxel in combination with either 10 μM EMD87580 (Fig. 4A), or 10 nM HMA (Fig 4B). This effect was significant when compared to cells treated with paclitaxel, EMD87580, or HMA alone at the same concentrations. Similar results were observed in MDA-MB-468 cells (P<0.001, N=5), but not in MCF7 cells. In contrast, in unstimulated 231-WT cells, viability was not further reduced by the combination of paclitaxel and NHE inhibitors (not shown). Likewise, in 231-KO cells, no significant differences were observed between treatments; however, treatment with 1 nM paclitaxel alone slightly but significantly reduced viability (P<0.05, N=5).

### NHE1 inhibition enhances the effect of paclitaxel on cell migration of triple-negative breast cancer cells

**Figure 5:**
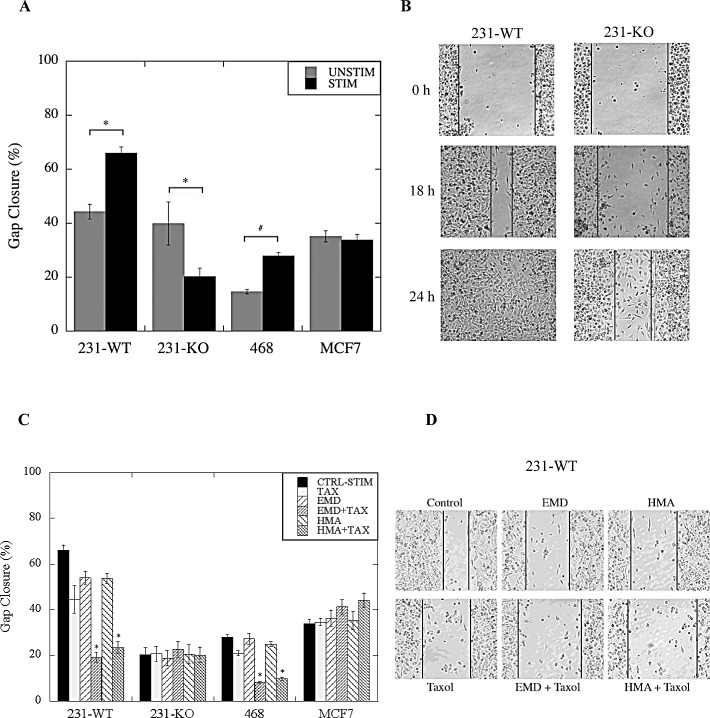
Effect of paclitaxel in combination with NHE1 inhibitors on cell migration of wild type (WT) and NHE1-knockout (KO) MDA-MB-231, MDA-MB-468 (468) and MCF7 cells The rate of closure of an induced gap was evaluated using a qualitative wound-healing assay as described in the Materials and Methods. A, Rate of gap closure in stimulated (STIM) (0.2% serum) and unstimulated (UNSTIM) (10% serum) 231-KO cells relative to 231-WT cells, and in comparison to MDA-MB-468 and MCF7 cells. Stimulated 231-WT and MDA-MB-468 cells migrate faster than unstimulated cells [*P<0.001, ^#^ P<0.05, N=10]. In contrast, in 231-KO cells, faster migration is observed in unstimulated conditions [*P<0.001, N=10]. B, Pictorial representation of gap closure (at 10X magnification) in serum-deprived 231-WT and 231-KO cells over time. C, Combined effect of paclitaxel and NHE1 inhibitors on the rate of migration. 1 nM paclitaxel (TAX) was evaluated in combination with either HMA (10 nM) or EMD87580 (10 μM). Arbitrary measurements of gap closure (normalized to the untreated controls) were pooled over multiple independent experiments and quantified with Image Pro Plus software [*P<0.001, ^+^ P<0.01, ^#^ P<0.05, N=10]. D, Pictorial representation of gap closure (at 10X magnification) in serum-deprived 231-WT cells treated with paclitaxel, either alone or in combination with EMD87580 or HMA at 18 hr.

We next examined the effect of NHE1 inhibition, in combination with paclitaxel, on cell migration in the three breast cancer cell lines. Stimulated (0.2% serum) 231-WT cells, migrated at a rate approximately 20% faster than unstimulated (10% serum) cells at 18 hr. post-scratch (P<0.001, N=10, Fig. 5A, B), whereas stimulated MDA-MB-468 cells migrated at about 10% faster than unstimulated cells (P<0.01, N=10). No differences were observed in MCF7 migration between stimulated and unstimulated cells. Interestingly, unstimulated 231-KO cells migrated faster than stimulated cells (P<0.05, N=10), a reversal of what is observed with the 231-WT. There was no difference in the rate of migration between unstimulated parental and NHE1-knockout MDA-MB-231 cells. Notably, when stimulated 231-WT or MDA-MB-468 cells were treated with paclitaxel in combination with either NHE1 inhibitor EMD87580 or HMA, a marked decrease in migration was observed (P<0.001, N=10, Fig. 5C, D). However, no such effect of drug treatments was seen in stimulated 231-KO or MCF7 cells, or in any cell type in unstimulated conditions (not shown).

### NHE1 promotes cell invasiveness and its inhibition potentiates effects of paclitaxel on cell invasiveness

**Figure 6:**
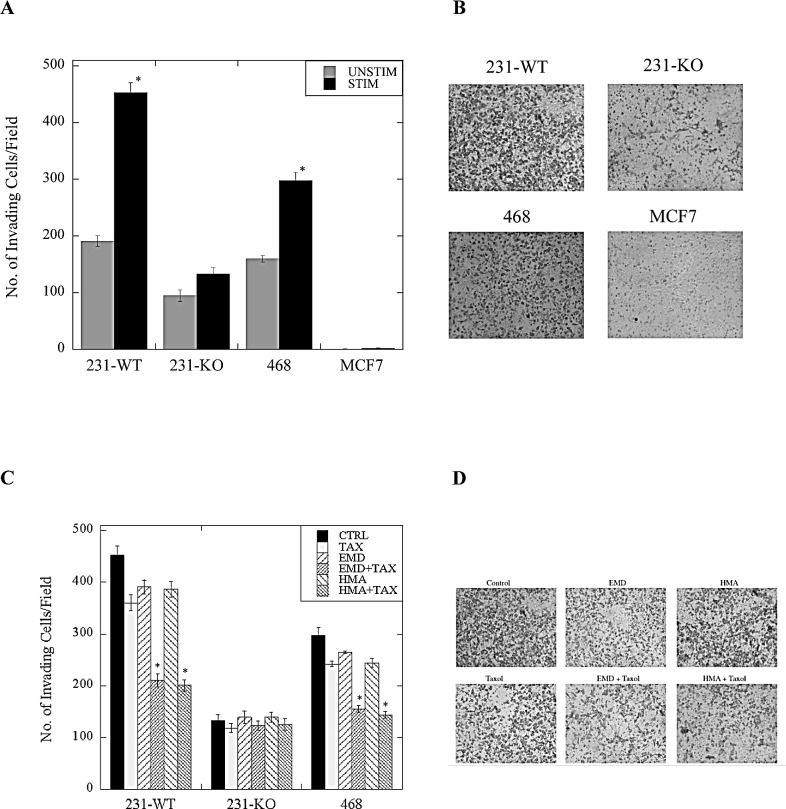
Invasiveness of stimulated (0.2% serum) or unstimulated (10% serum) 231-WT, 231-KO, MDA-MB-468 or MCF7 cells treated with paclitaxel +/− NHE1 inhibitors The rate of cell invasion was determined using a Matrigel-coated Boyden chamber porous insert as described in the Materials and Methods. A, Invasiveness of 231-KO cells, +/− stimulation, relative to 231-WT cells, and in comparison to MDA-MB-468 (468) and MCF7 cells [*P<0.001, N=4]. Side panel (B) illustrates representative images. C, Effect of paclitaxel (1 nM, TAX) in combination with NHE1 inhibitors (10 μM EMD87580 or 10 nM HMA) on cell invasion in wild type and NHE1-knockout MDA-MB-231, and MDA-MB-468 cells [*P<0.001, N=4]. D, Illustration of invasion of stimulated 231-WT cells.

Cell invasion assays were performed to gain insights into the role of NHE1 in invasion and metastasis of the various types of breast cancer cells. Cell invasion was significantly reduced in 231-KO cells compared to 231-WT parental cells cultured in either stimulated (0.2% serum) or unstimulated (10% serum) conditions for 24 hr. prior to invasion. Additionally, stimulation of cells by reduction of serum caused invasion to more than double in 231-WT cells, but had no effect on 231-KO cells (P<0.001, N=4, Fig. 6A, B). A similar marked increase in invasive potential was seen in another triple-negative breast cancer cell line, MDA-MB-468 cells, with stimulation by reduced serum. No detectable invasion was observed in MCF7 cells in this assay.

We also determined the effect of NHE1 inhibition in combination with paclitaxel. When stimulated 231-WT, 231-KO or MDA-MB-468 cells were treated with paclitaxel in the presence or absence of NHE1 inhibitors, EMD87580 or HMA, we found a significant reduction in the number of invading 231-WT and MDA-MB-468 cells after 24 hours (P<0.001, N=4, Fig. 6C, 6D), but no effect on 231-KO cells.

**Figure 7:**
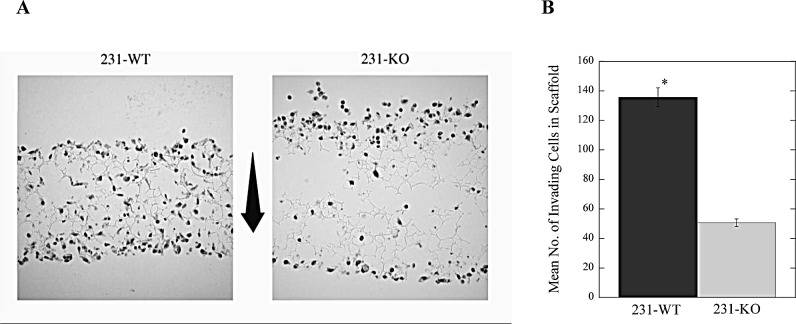
Long-term invasion is dependent on the expression of NHE1 in MDA-MB-231 breast cancer cells A, Representative image of 231-WT and 231-KO cells in 3-dimensional scaffold over 7 days in 10% serum; arrow shows the direction of invasion from top to bottom of the scaffold. B, Summary of results showing the number of invading cells [*P<0.001, N=3].

Finally, we examined the role of NHE1 in a study of 3-dimensional long-term invasion by breast cancer cells. Cells were seeded onto a Matrigel matrix-coated scaffold that enabled 3-dimensional growth and proliferation, mimicking cell growth and association with the extracellular matrix *in vivo*. NHE1 knock out cells (231-KO) cells did not survive in serum-deprived (0.2% serum) conditions for 7 days in this assay, so the analysis was limited to serum-supplemented conditions. Here, 231-WT cells grown in 10% serum showed significantly more invasion through and, greater proliferation within, the scaffold than 231-KO cells (Fig. 7).

## DISCUSSION

Both established and novel chemotherapeutic strategies can have limited success in the treatment of metastatic cancer, especially due to the development of multidrug resistance [23]. This is particularly apparent in the treatment of triple-negative breast cancer, which is negative for the expression of estrogen and progesterone receptors and HER2, and therefore not susceptible to hormone or HER2-targeted therapy. Experimentation has shown that enhanced NHE1 activity acidifies the extracellular pH, and promotes metastasis of highly invasive types of breast cancer cells [24-27]. The phenomenon of NHE1 activation and acidification promoting tumorigenesis and invasiveness is not specific to breast cancer and also occurs in various forms in malignant melanoma [28], in fibrosarcoma cells [29], and in human colon cancer cells [29]. It stands to reason, therefore, that manipulating the tumor microenvironment through the modulation of NHE1 activity could augment chemotherapy strategies and potentially lessen the incidence of multidrug resistance. This approach might prove useful wherever multidrug resistance occurs, in both triple negative and non-triple negative breast cancers.

In the present study, we therefore investigated the role of NHE1 in breast cancer cell invasion, migration and in tumor formation. Additionally, we studied the role of NHE1 in susceptibility to paclitaxel, one of the most effective chemotherapy drugs used in the treatment of this disease. These studies represent the first work that utilizes a complete, stable, and functional knockout of NHE1 in metastatic MDA-MB-231 breast cancer cells. Unlike transient silencing of NHE1 with targeted siRNA or partial knockdown of NHE1 with targeted shRNA, we constructed a complete functional knockout of NHE1 in MDA-MB-231 breast cancer cells using zinc finger nucleases, thus enabling both *in vivo* and *in vitro* analysis of the physiological role of the Na^+^ /H^+^ exchanger.

We characterized the role of NHE1 in culture conditions either supplemented with serum or serum deprived. Serum deprivation occurs *in vivo* in the tumor microenvironment and tumor cells respond differently than normal cells to low-serum culture conditions. For example, NHE1 becomes constitutively active when serum is depleted [30, 31]. We found that the relative Na^+^ /H^+^ exchange activity in stimulated 231-WT cells was markedly higher compared to unstimulated cells, where a basal NHE1 activity was observed. We further demonstrated that this elevated NHE1 activity in stimulated cells was not due to an increased expression of the NHE1 protein. Instead, it must have been due to changes in the regulation of NHE1 consistent with earlier observations [32]. Serum deprivation reduces lactate production in both non-tumorigenic (MCF10A) and tumorigenic (MCF7) breast epithelial cell lines, but while it inhibits NHE1 activity in the non-tumorigenic cells, depletion of serum stimulates exchanger activity in tumor cells. This was earlier reported to be dependent on phosphoinositide-3-kinase (PI3K), though it was not known if PI3K phosphorylates NHE1 directly or through some unknown intermediary [30]. Later observations indicated that serum deprivation activates NHE1 in breast cancer cells via a sequential RhoA/p160ROCK/p38MAPK signaling pathway, gated by direct protein kinase A phosphorylation and concurrent inhibition of RhoA [33].

Elevated activity of NHE1 in tumor cells can affect intracellular ion concentrations. *In vivo*, the activity of Na^+^ transporters would result in cellular alkalinization that is typical of tumor cells [34]. This results in a concomitant increase in sodium loading. In cardiomyocytes [35], and malignant gliomas [36], this increased NHE1 activity and elevated intracellular Na^+^ causes elevated intracellular Ca^2+^ through reversed activity of the Na^+^ /Ca^+2^ exchanger [37]. How important this pathway is in tumor cells is unclear at this time, however, it is known that two important protein regulators of NHE1, calmodulin and CHP (calcineurin regulatory protein) are calcium dependent in their association. One isoform of CHP, CHP2 is upregulated in tumor cells and the CHP2-NHE1 interaction is suggested to be key in maintenance of the cellular alkalinization associated with malignantly transformed cells [38]. Further studies are ongoing to characterize the complex regulation of NHE1 in breast cancer cells.

When we examined the role of NHE1 in triple-negative breast cancer cells overall, our results showed that NHE1 has a facilitative role in breast cancer invasion and metastasis, as well as a chemo-protective role in highly invasive triple-negative breast cancer cells. These metastasis-enhancing roles were not apparent in less invasive, non-triple-negative, breast cancer cells. The evidence for this statement comes from experiments both *in vivo* and *in vitro*, and from experiments in which we knocked out the NHE1 protein or inhibited its activity. In summary, the experiments suggesting that NHE1 facilitates metastatic breast cancer are: 1, knockout of NHE1 reduced the *in vivo* tumor-promoting capability of MDA-MB-231 cells; 2, NHE1 deletion in MDA-MB-231 cells reduced their invasive capacity; and 3, specific inhibition of NHE1 potentiated the effect of paclitaxel, reducing cell viability, invasion and migration. These are discussed below.

*1, NHE1-Knockout*: Knocking out NHE1 from the highly tumorigenic MDA-MB-231 cells significantly reduces their tumor-promoting capability *in vivo*. This was demonstrated by the differences in subcutaneous growth of tumor xenografts of parental and knockout cells in athymic nude mice. Interestingly, despite comparable rates of proliferation between the parental and NHE1-knockout cells *in vitro*, little or no tumor development was apparent in xenografts of 231-KO cells. These results are the first such demonstration of the critical role of NHE1 in tumor development of triple-negative breast cancer cells *in vivo*. In this study, we also demonstrated that in an *in vitro* invasion assay, NHE1-knockout cells were unable to survive serum-depleted conditions over seven days. This might account for their inability to promote tumor growth *in vivo*.

*2, Loss of NHE1 reduces cell migration and invasion of triple-negative breast cancer cells*: Knockout of NHE1 from MDA-MB-231 cells dramatically decreased the migratory capacity of these cells, an effect that is independent of their rates of proliferation over 18 hours. In addition, the invasive potential of these normally aggressively invasive breast cancer cells was markedly reduced with the deletion of NHE1. Even in the presence of serum, NHE1-knockout cells were significantly less invasive through an extracellular matrix than parental cells, particularly over several days, as shown by the 3-dimensional invasion assay data. This confirms that NHE1 is critical to the molecular mechanisms involved in invasion. Notably, while the viability of stimulated 231-WT cells was unaffected by increasing doses of paclitaxel, deletion of NHE1 in the knockout cells (231-KO) made them more susceptible to paclitaxel-mediated cell death (at doses upwards of 0.1 nM), suggesting a chemo-protective role for NHE1 in MDA-MB-231 cells.

*3, Specific inhibition of NHE1 potentiates effects of paclitaxel*: We found that two structurally different NHE1 inhibitors, EMD87580 [(2-methyl-4,5-di-(methylsulfonyl)-benzoyl)-guanidine], and HMA [5-(N, N-hexamethylene)-amiloride] specifically potentiated effects of paclitaxel on triple-negative breast cancer cells. This included effects on cell migration, viability and invasion when the combinatorial treatment of paclitaxel in the presence of NHE1 inhibitors was applied to MDA-MB-231 cells. Here, we found that paclitaxel alone caused a slight but significant decrease in the migration of stimulated triple-negative MDA-MB-231 and MDA-MB-468 breast cancer cells. However, when paclitaxel was used in the presence of NHE1 inhibitors, its effect was greatly enhanced. Likewise, paclitaxel, EMD87580, and HMA alone, all had an effect on the invasiveness of these cells when stimulated, however, when paclitaxel was used in combination with an NHE1 inhibitor, the reduction in the rate of invasion was much more pronounced. These effects did not occur in non-triple-negative MCF7 breast cancer cells.

Our results are a novel finding with human breast cancer cells. While one study has shown that the amiloride analogue DMA, (5-(N,N-dimethyl) amiloride-hydrochloride) potentiates paclitaxel-mediated apoptosis [39], it is now known that MDA-MB-435 are in fact a melanoma cell line [40]. Nevertheless, those results support the idea that NHE1 inhibition can enhance the effects of paclitaxel on cancer cells. The concept of inhibiting NHE1 activity as a potential strategy to augment existing chemotherapeutic treatment of tumor cells, and in the management of tumor size, is intriguing. Our experimental design used NHE1 inhibition with paclitaxel treatment at doses much lower than the established IC_50_ of 2.4 nM for paclitaxel in MDA-MB-231 cells [41]. The beneficial effects did not occur in unstimulated cells, where the rate of NHE1 exchanger activity is minimal. It therefore appears that this combinatorial treatment effect may be contingent upon NHE1 hyper-activation, as occurs when cells are serum-deprived in the tumor microenvironment. Remarkably, these effects were also observed in the moderately invasive, triple-negative, basal-like MDA-MB-468 breast cancer cells, albeit to a lesser extent. In contrast, we did not observe a similar trend with the lowly invasive, hormone receptor-positive, luminal MCF7 cells, at least with the drug concentrations used in the present study. These data suggest that the observed effects may be specific to triple-negative breast cancers which have a constitutively activated NHE1 protein in the disease state [32].

The potential of NHE1 as a target in the development of novel anti-cancer therapeutics was recently reviewed, highlighting key characteristics of NHE1 that could be exploited to combat the tumorigenic and metastatic capacity of cancer cells [42]. The NHE1 gene, *SLC9A1* is a redox regulated gene. Down-regulation of NHE1 expression decreases the capacity of cells to recover from an acute acid load and sensitizes them to cell death triggers [43]. One approach to down-regulation of NHE1 may be through PPAR (peroxisome proliferator-activated receptor). A peroxisome proliferator response element was identified in the promoter region of the NHE1. Expression of PPARγ (peroxisome proliferator-activated receptor γ) is increased in breast cancer cell lines and primary breast tumors, and correlates with an acidic intracellular pH and it was suggested that judicious use of PPARγ ligands at low doses could have significant anti-cancer effects in combination with chemotherapy [44]. Whether or not the anti-cancer effects of down-regulation of NHE1 gene expression are comparable to the chemical inhibition of NHE1 activity, has yet to be explored in depth.

In this study, we suggest that NHE1 inhbition may reduce the acidic extracellular pH of the tumor microenvironment, thereby rendering paclitaxel more effective. Could inhibition of NHE1 *in vivo* also improve the efficacy of paclitaxel treatment? This idea is supported by evidence suggesting that another NHE1 inhibitor, amiloride-HCl, though it has low NHE1 specificity, still might have anti-cancer properties [14]. Certainly, NHE1 inhibitors have been used in clinical trials involving heart disease, however, there are still challenges left before their use is approved [45]. Possibly, the development of more selective NHE1 inhibitors, or inhibitors that specifically target tumor cells, might be productive in the treatment of breast cancer. The more potent of new highly selective NHE1 inhibitors includes: cariporide, (N-(Diaminomethylene)-4-isopropyl-3-(methylsulfonyl)benzamide); the phenoxazine derivative Phx-3 (2-aminophenoxazine-3-one); and Compound 9t (5-aryl-4-(4-(5-methyl-1H-imidazol-4-yl)piperididn-1-yl)pyrimidine analog). The efficacies and specificity for NHE1 inhibition of these compounds and their potential for use in future clinical and/or pre-clinical trials should be considered for the treatment of triple-negative breast cancer, as well as for BRCA1-positive breast cancer. Indeed, the use of selective NHE1 inhibitors can be applied to the treatment of other human malignant tumors in attempts to not only prevent tumor formation, but also circumvent multidrug resistance [16, 27].

Since pH regulation plays an integral role in the tumor microenvironment, it is highly plausible to consider the modulation of Na^+^ /H^+^ exchanger activity as a means of increasing the efficacy of chemotherapy, particularly for triple-negative breast cancer, for which targeted therapies do not currently exist. Realistically, this synergistic approach using NHE1 inhibitors as co-adjuvants to established chemotherapeutic drugs, could arguably be effective in augmenting the targeted treatment of the HER2-positive subtype of breast cancer as well. Future experiments could address this in multiple breast cancer subtypes. Moreover, modulating the pH of the tumor microenvironment could conceivably decrease the incidence and spread of multidrug resistance for anti-cancer agents in general. Overall, our data highlight a novel putative, clinical role for NHE1 inhibitors targeted to modulate pH in the tumor microenvironment and to maximize the efficacy of breast cancer chemotherapy.

## MATERIALS AND METHODS

### Cell lines and culture conditions

Parental MDA-MB-231 (231-WT), 231-KO (NHE1-knockout), MDA-MB-468 and MCF7 cells were cultured in high-glucose modified DMEM (HyClone) supplemented with 10% fetal calf serum (HyClone), 10 mM HEPES, and 1000 units/ml penicillin/streptomycin (Gibco) under standard culture conditions (5% CO_2_, 37°C and high humidity). Starvation media was supplemented with 0.2% serum but otherwise identical in composition. Paclitaxel (Taxol®, Sigma) and NHE1 inhibitors, EMD87580 [2-methyl-4,5-di-(methylsulfonyl)-benzoylguanidine, gift from Merck] and HMA [5-(N, N-hexamethylene)-amiloride, Sigma], were used at indicated concentrations. MDA-MB-231, MDA-MB-468 and MCF7 cell lines were authenticated by DNA analysis (DDC Medical, Ohio) and showed >95% homology to the ATCC STR profile.

### Generation of 231-KO (NHE1-knockout) cells

In MDA-MB-231 cells, endogenous NHE1 was excised using CompoZr® Knockout Zinc Finger Nucleases (Sigma-Aldrich) specifically designed to target the human NHE1 gene *SLC9A1*, and according to the manufacturers' protocols. Potential knockout cells were screened by western blot using anti-NHE1 antibody (BD Transduction Laboratories) and re-selected using the proton suicide assay as described earlier [46]. Briefly, cells are incubated in a Na^+^-free lithium chloride (LiCl) buffer for two hours to allow for NHE1-mediated uptake of Li^+^ in the absence of Na^+^, which results in acidification of the extracellular pH. LiCl buffer is then replaced with choline chloride buffer for one hour. NHE1 is unable to transport choline, resulting in the reversal of proton exchange across the plasma membrane and acidification of intracellular pH. Intracellular proton accumulation becomes lethal in cells with a functional exchanger whereas NHE1-knockout cells survive, thereby ensuring the purity of the 231-KO cell line.

### Xenograft tumor growth experiments

All animal experiments/husbandry were approved and follow the guidelines of the Canadian Animal Care and Use Committee. Subcutaneous injection of tumor cells was carried out as previously described [47]. Parental MDA-MB-231 and NHE1-knockout (231-KO) cells were grown to confluence in 100 mm^2^ culture dishes, washed in phosphate-buffered saline (PBS), trypsinized, and resuspended in complete media. After centrifugation at 1000 rpm for 5 min, cells were washed once in PBS and resuspended in a 4:1 mixture of serum-free media:Matrigel (BD #354234, 10 mg/ml of LDEV-free matrix). Two hundred microliter (containing ~ 2.5 × 10^6^ cells) of cells:Matrigel suspension was subcutaneously injected into the right and left dorsal flanks of female athymic nude mice (Taconic Laboratories; #NCRNU-F, CrTac:NCr-FoxN1Nu) to determine the tumor-promoting potential of the 231-WT and 231-KO breast cancer cells. Mice were monitored weekly to assess tumor growth and euthanized at Day 60. Tumor volume was calculated based on the volume of a sphere (V = [4/3)πr^3^ ] or the length and width of the tumor mass (V = 0.5*x* X 2*y*, where *x* is the length and *y* is the width of the tumor mass) wherever appropriate.

### Western blotting

Total protein from whole cell lysates was separated by 10% SDS polyacrylamide gels and transferred onto nitrocellulose membranes. Blots were incubated overnight with anti-NHE1 antibody (BD Transduction Laboratories) and anti-actin antibody (Santa Cruz Biotechnologies) was used as a loading control. Image J software was used to quantify NHE1 expression.

### Na^+^ /H^+^ exchange activity (pHi) assay: Intracellular pH measurement

The pH-sensitive dye BCECF-AM (2′,7′-bis(carboxyethyl)-5(6)-carboxyfluorescerin-acetoxymethyl ester) was used to measure intracellular pH (pH_i_) as described earlier [48]. Briefly, cells were grown to 80% confluence on rectangular glass coverslips (22 × 11 mm) in 35 mm dishes in complete media. Once attached, cells were either serum-starved (stimulated) or not (unstimulated) for 24 hr. prior to loading with BCECF-AM for 20 minutes at 37°C. BCECF-AM is a cell permeable, non-fluorescent dye that is de-esterified to BCECF upon entry into cells. *In cellulo*, BCECF fluoresces in response to changes in intracellular pH. After incubation with BCECF, followed by incubation in a Na^+^-containing equilibration buffer for 3 min, an acute acid load is induced by treating cells with ammonium chloride (50 mM × 3 min). After removal of NH_4_Cl, followed by reperfusion with Na^+^-free and Na^+^-containing buffers, cells are placed in Na^+^-free calibration buffer containing nigericin and high K^+^ to calibrate signal ratios at pH 6, 7 and 8. The rate of recovery of pH_i_ as indicated by the ratio of BCECF fluorescence post-excitation at 440 nm and 502 nm, and emission at 528 nm, was recorded using a PTI Deltascan Illumination System (Photon Technology International, New Jersey, USA). NHE1 activity was calculated from the slope of the first 20 sec of recovery from acidification and was expressed as ΔpH/sec. Background drift in the absence of Na^+^ was subtracted from rates of recovery in the presence of Na^+^, and all data were normalized to controls to show relative Na^+^ /H^+^ exchange activity between cell types. Buffering capacity was calculated and shown to be similar across all cell types.

### Cytotoxicity, Viability and Proliferation assays

Cells were seeded in 96-well plates at a density of 1 × 10^5^ cells/well, and left untreated (control) or treated with paclitaxel and/or NHE1 inhibitors EMD87580 or HMA in phenol red-free high-glucose modified DMEM (HyClone) media supplemented with either 10% (unstimulated cells) or 0.2% (stimulated cells) fetal calf serum and incubated for 0 to 48 hr. under standard culture conditions. Unless otherwise noted, drug concentrations were used as follows: paclitaxel (1 nM), EMD (10 μM) and HMA (10 nM). MTT [3-(4,5-dimethylthiazol-2-yl)-2,5-diphenyltetrazolium bromide, Sigma] assays with a final MTT concentration of 0.5 mg/ml were used to assess cytotoxicity to drugs, resultant cell viability (at 24 hr.) and proliferation (at 0, 24, 48 hr.). Absorbance was measured with a BioTek Synergy MX microplate reader (BioTek Instruments Inc.) at 570 nm and background (at a reference wavelength of 630 nm) was subtracted. All viability data were normalized to untreated controls. Proliferation data was represented as a measure of the increase in the rate of cell proliferation from time 0 hr.

### Migration assay

Cell migration was studied using the qualitative wound-healing assay. Briefly, cells were seeded in 24-well plates (0.5 × 10^6^ cells/well), and grown to confluence, before wounding was induced with a pipette tip. Cells were washed with PBS to remove unattached cells, supplemented with media, and either left untreated (control), or treated with paclitaxel and/or NHE1 inhibitors with either 10% (unstimulated cells) or 0.2% (stimulated cells) serum and incubated for 0 to 24 hr. Images were acquired at 0, 18 and 24 hr. time-points. Arbitrary measurements of gap closure were quantified with Image Pro Plus software using a Leica DM IRB microscope (at 10X magnification). A minimum of three images/well and five measurements/image was acquired for each treatment; all treatments were done in triplicate. Data shown are a representation of decrease in gap distance at 18 hr. compared to the initial measurement at 0 hr over multiple independent experiments.

### Invasion assays

Cell invasion assays were performed using Boyden chamber 8 μm porous inserts (Corning) coated with Matrigel (BD Biosciences) according to the manufacturer's recommendations. Briefly, cells were either serum-starved (stimulated) or not (unstimulated) and/or treated with appropriate drugs for 24 hr. prior to adding 1 × 10^5^ cells per insert. Reservoir media contained 10% serum to promote chemotaxis. After 24 hr., cells on the upper surface of the insert were removed with a cotton swab and invading cells on the lower surface were fixed in 3.7% paraformaldehyde, permeabilized in 100% methanol, and stained with Giemsa. Invasion was a measure of how many cells traversed the membrane, and was imaged at 10X magnification with Image Pro Plus software using a Leica DM IRB microscope. Data shown are a representation of 3 to 5 fields/insert over multiple independent experiments. 3D invasion assays were carried out with 6-well Alvetex^®^ scaffold inserts (Reinnervate, UK), which were coated with Matrigel according to manufacturer's instructions. Briefly, inserts were pre-treated with 70% ethanol to prime scaffolds for cell culture. After washing in PBS, scaffolds were coated with 0.8 mg/ml of Matrigel prior to adding cells. Cells were seeded at a density of 1 × 10^6^ cells in 100 μL of media per insert and allowed to attach to the scaffold surface for 90 minutes prior to adding media. Serum supplemented media (10%) was added for the first 24 hr. to facilitate initial cell adhesion. After 24 hr., media was changed and cells were either serum-starved (stimulated, 0.2% serum) or not (unstimulated, 10% serum). Cells were allowed to invade through the scaffold over 7 days with media changes every 2 days. Scaffold discs were then excised from the insert, embedded in paraffin, and longitudinally sectioned into multiple 10 μm sections prior to staining with hematoxylin and eosin. Invasion was a measure of how many cells traversed the scaffold, and was imaged at 20X magnification with a Zeiss ApoTome Microscope and Zeiss AxioCam MRm imaging software. Data shown are a representation of triplicate sections per slide with two slides per treatment of each scaffold disc, and three independent experiments.

### Statistical Analysis

All data are expressed as means ± SEM and plotted with KaleidaGraph 4.1 (Synergy Software, PA, US). Data shown are a mean of N=3 to 10 independent experiments. Statistical analysis was carried out using the two-way analysis of variance (ANOVA) to compare data between groups with GraphPad Prism 5.0 (GraphPad Software, CA, US). Post hoc comparisons were made using Bonferroni's multiple-comparison tests. A P value less than 0.05 was considered to be signiﬁcant.

## Abbreviations

231-KO: 231-knock out
231-WT: 231-wild type
BCECF-AM: 2′,7′-bis(carboxyethyl)-5(6)-carboxyfluorescerin-acetoxymethyl ester
CHP1/2: Calcineurin homologous protein 1/2
EMD87580: (2-methyl-4,5-di-(methylsulfonyl)-benzoyl)-guanidine)
ERK1/2: Extracellular signal-regulated kinases 1/2
ERM: Ezrin-radixin-moesin
HER2: Human epithelial growth factor receptor 2
HMA: 5-(N, N-hexamethylene)-amiloride
MAPK: Mitogen-activated protein kinases
MTT: 3-(4,5-dimethylthiazol-2-yl)-2,5-diphenyltetrazolium bromide
NHE1: Na^+^ /H^+^ exchanger isoform 1
PIP_2_: Phosphatidylinositol biphosphate
TAX: Paclitaxel (Taxol^®^)
